# Retraction Note: Dioscin induces prostate cancer cell apoptosis through activation of estrogen receptor-*β*

**DOI:** 10.1038/s41419-021-04083-y

**Published:** 2021-08-20

**Authors:** Xufeng Tao, Lina Xu, Lianhong Yin, Xu Han, Yan Qi, Youwei Xu, Shasha Song, Yanyan Zhao, Jinyong Peng

**Affiliations:** College of Pharmacy, Dalian Medical University, Western 9 Lvshunnan Road, Dalian, 116044 China

Retraction Note: *Cell Death & Disease* 10.1038/cddis.2017.391, published online 10 August 2017

The Editors-in-Chief have retracted this article because concerns have been raised regarding some of the figures. An investigation by the College of Pharmacy Dalian Medical University has established that during the course of revision, the result of the transwell migration assay in Fig. 5c was provided by mistake due to file placement confusion. In Fig. 7c, the graph of HE staining of ERβ-siRNA+Dioscin group was incorrectly uploaded and was different from our original data. While the institutional investigation has not found evidence of misconduct, the Editors-in-Chief have found that due to the errors the findings are no longer reliable. The authors all agree with this retraction.

